# Precision and Advanced Nano-Phytopharmaceuticals for Therapeutic Applications

**DOI:** 10.3390/nano12020238

**Published:** 2022-01-12

**Authors:** Chooi Ling Lim, Chandramathi S. Raju, Tooba Mahboob, Sunil Kayesth, Kamal K. Gupta, Gaurav Kumar Jain, Mahaveer Dhobi, Muhammad Nawaz, Polrat Wilairatana, Maria de Lourdes Pereira, Jayanta Kumar Patra, Alok K. Paul, Mohammed Rahmatullah, Veeranoot Nissapatorn

**Affiliations:** 1 Division of Applied Biomedical Science and Biotechnology, School of Health Sciences, International Medical University, Kuala Lumpur 57000, Malaysia; 2 Department of Medical Microbiology, Faculty of Medicine, University of Malaya, Kuala Lumpur 50603, Malaysia; tooba666@hotmail.com; 3 Department of Zoology, Deshbandhu College, University of Delhi, New Delhi 110019, India; kgupta@db.du.ac.in; 4 Department of Pharmacognosy and Phytochemistry, Delhi Pharmaceutical Sciences and Research University (DPSRU), New Delhi 110017, India; gkjain@dpsru.edu.in (G.K.J.); mahaveer@dpsru.edu.in (M.D.); 5 Department of Nano-Medicine, Institute for Research and Medical Consultations ((IRMC), Imam Abdulrahman Bin Faisal University, Dammam 34212, Saudi Arabia; mnnmuhammad@uod.edu.sa; 6 Department of Clinical Tropical Medicine, Faculty of Tropical Medicine, Mahidol University, Bangkok 10400, Thailand; 7 CICECO-Aveiro Institute of Materials & Department of Medical Sciences, University of Aveiro, 3810-193 Aveiro, Portugal; mlourdespereira@ua.pt; 8 Research Institute of Biotechnology & Medical Converged Science, Dongguk University-Seoul, Goyang-si 10326, Korea; jkpatra@dongguk.edu; 9 School of Pharmacy and Pharmacology, University of Tasmania, Private Bag 26, Hobart, TAS 7001, Australia; alok.paul@utas.edu.au; 10 Department of Biotechnology & Genetic Engineering, University of Development Alternative, Lalmatia, Dhaka 1207, Bangladesh; rahamatm@hotmail.com; 11 School of Allied Health Sciences and World Union for Herbal Drug Discovery (WUHeDD), Walailak University, Nakhon Si Thammarat 80160, Thailand

**Keywords:** nano-formulations, phytopharmaceuticals, herbal medications, therapeutics, precision

## Abstract

Phytopharmaceuticals have been widely used globally since ancient times and acknowledged by healthcare professionals and patients for their superior therapeutic value and fewer side-effects compared to modern medicines. However, phytopharmaceuticals need a scientific and methodical approach to deliver their components and thereby improve patient compliance and treatment adherence. Dose reduction, improved bioavailability, receptor selective binding, and targeted delivery of phytopharmaceuticals can be likely achieved by molding them into specific nano-formulations. In recent decades, nanotechnology-based phytopharmaceuticals have emerged as potential therapeutic candidates for the treatment of various communicable and non-communicable diseases. Nanotechnology combined with phytopharmaceuticals broadens the therapeutic perspective and overcomes problems associated with plant medicine. The current review highlights the therapeutic application of various nano-phytopharmaceuticals in neurological, cardiovascular, pulmonary, and gastro-intestinal disorders. We conclude that nano-phytopharmaceuticals emerge as promising therapeutics for many pathological conditions with good compliance and higher acceptance.

## 1. Introduction

Nanotechnology is the application of nanoparticles for various purposes, including disease diagnostics, cosmetics, and therapeutics. From a small beginning in the 1950s by the physicist Richard Feynman and in 1974 by Norio Taniguchi, the applications of nanotechnology have changed to an enormous dimension today, and are encompassing many branches of chemistry, physics, biochemistry, biotechnology, information technology, environmental science, and pharmacy, to name only a few [1]. Nanotechnology is a vital tool for medical sciences. The introduction of nanomedicine, using nanotechnology combined with drugs or diagnostic molecules, has improved the ability to target specific cells or tissues that require treatment and repair. These nanomaterials have been proven to be produced at a nanoscale level and are safe to introduce into the body. It is possible to modify a variety of nanocarriers’ characteristics that include their constituents, size, shape, bioavailability, surface properties, and target specificity to achieve or enhance desirable pharmacological targets [1,2] Several strategies have been implemented to increase the drug-target specificity. Recently, several studies have reported improved efficacy of therapy when combined with nanomaterials [3]. Pure herbal medicines are often considered less effective compared to pure constituents that are mainly demonstrated to have reduced intestinal absorption when administered orally [4]. This is the reason behind the pharmacological activity/loss associated with pure constituents and such problems can be overcome using new drug delivery systems, such as nanotechnology.

Nanoparticulate delivery of drugs can generally improve drugs’ solubility, bioavailability, stability, pharmacological activity, increase target specificity, promote transport across membrane, prolong circulation times, and reduce systemic and organ toxicity [5,6]. Various treatments are being investigated with the use of nanoparticle drug delivery systems for diseases, such as infectious diseases, autoimmune diseases, cardiovascular diseases, neurodegenerative diseases, ocular diseases, fungal infections, iron deficiency, and pulmonary diseases [7,8]. However, the greatest advances were seen in the treatment of cancer with several nano strategies being used clinically after approval by the FDA in the United States of America [9]. Recently, more attention has shifted towards novel drug delivery systems using nanoparticles for herbal and plant-based drugs [10]. The use of plants for medicinal purposes can be documented from as far back as 6000 years ago with many important plant compounds being developed into conventional or allopathic medicine used today [11]. Although conventional medicine is widely used compared to herbal medicine in developed countries, there is a considerably large segment of society that continues to use medicines derived from plants for their healthcare [12].

According to the World Health Organization, nine million Malaysians out of an estimated population of 30 million would have used or are using traditional and complementary medicines (which includes plants, parts of plants, or plant materials) for the prevention and treatment of medical ailments. Additionally, 88% of WHO member states (170 of the 194 member states) have acknowledged their use of traditional and complementary medicine amongst their population [13].

**Figure 1:**
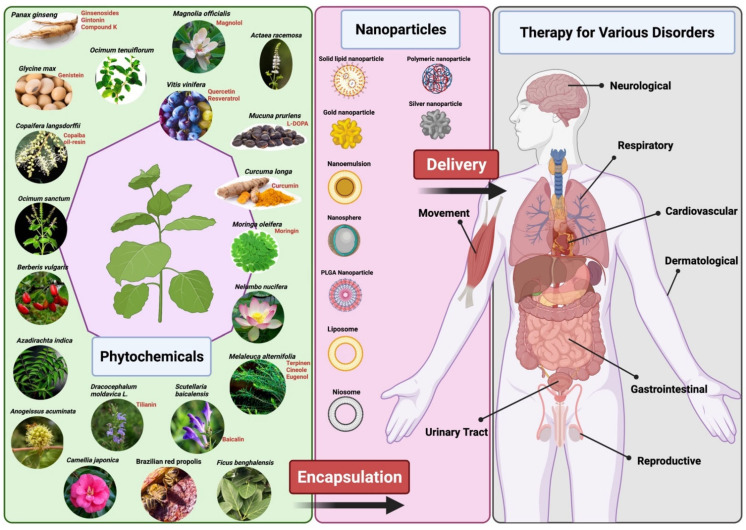
Representation of delivery of phytopharmaceuticals using nanotechnology. The figure was made with www.biorender.com (access date: 17 December 2021).

Despite these numbers, plant-based medicine has some limitations which hinder its use and production in the mainstream disease treatment and therapy. There are several chemical constituents in a plant’s extract that lead to its medicinal properties. The active constituents of plant extracts like tannins, flavonoids, alkaloids, phenylpropanoids, and terpenoids are water-soluble but show poor absorption from their inability to cross lipid membranes and have large molecular sizes, which then results in low bioavailability and efficacy [5,12,14]. There are also concerns of safety due to the incompatibility of some plant extracts with other components in a drug formulation which can lead to undesirable effects [12]. High systemic clearance of these compounds also leads to low therapeutic levels in the blood resulting in no therapeutic effect [15]. Furthermore, poor reproducibility of in vitro effects in vivo prevents many plant-based medicines from clearing clinical trial phases [16]. Nanomedicine aims to overcome these limitations and to improve the delivery of plant-based medicines to treat various diseases. Nanoparticle drug delivery systems can potentially improve the stability, solubility, and bioavailability of encapsulated plant extracts, promote its movement across lipid membranes, and prolong its circulation, all while delivering the active constituent to a specific target site [5,6,12] (Figure 1). Liposomes, dendrimers, polymeric NCs, polymeric micelles, metallic NPs (magnetic, gold), SLNs, nanocapsules, nanospheres, and nanogels are some of the examples of nano-based drug delivery systems that are presently under investigation [10]. This review aims to show the potential of plant-based drugs to prevent and treat some of the communicable and non-communicable diseases. Although not exhaustive, this review highlights several examples where the application of nanotechnology proves to be effective.

## 2. Materials and Methods

Databases including PubMed, Medline, Scielo, Thomson Reuters ISI Web of Knowledge, and Science Direct were searched, combining the following keywords: “Nanotechnology”, “plant-based medicine”, “herbal nanoformulations”, “phytochemical-based nanoformulations”, and “nano-phytopharmaceuticals”. The available scientific literature within the last decade (2011–2021) was considered in this review.

## 3. Therapeutic Applications of Nano-Phytopharmaceuticals

### 3.1. Nano-Phytopharmaceuticals in Neurological (CNS) Disorders

The central nervous system is a complex environment—a vast network of neurons, astrocytes, microglia, and other supportive tissues buoyed in lubricating cerebrospinal fluid. The delicate balance between nerve connections, spinal cord, and the control center, the brain, is easily perturbed by exogenous insults and endogenous degenerative conditions. Debilitating disorders of the central nervous system (CNS) include Alzheimer’s disease (AD) [17] and variants of dementia such as Lewy body dementia (LBD), tumors such as neuroblastoma and glioblastoma [18], as well as dyskinesia-related conditions such as Parkinson’s disease (PD), Huntington’s disease (HD), and amyotrophic lateral sclerosis (ALS) [19]. Alzheimer’s disease is a type of neurodegenerative disease that is described by the formation of plaques with tangled neurofibers composed of amyloid and tau proteins. It is recognized as the most common type of dementia and the main risk factor is associated with age [20]. Parkinson’s disease is described by the deficiency of dopamine neurons in the substantia nigra. It has been associated with the aggregation of ubiquitinated synuclein in the dopaminergic neurons [20]. It is estimated that 1% of individuals over 60 years of age are affected by Parkinson’s disease, which is the main source of the movement disorder known as Parkinsonism [20]. Parkinsonism comprises tremors, bradykinesia, and akinesia in severe cases [21]. Huntington’s disease is described by the accumulation of a mutant Huntingtin protein in the neurons, prompting inaccurate neuronal actions and eventually results in neuronal demise [22]. This prompts unusual movements in the patient, adduced as chorea [23]. The cause of Huntington’s disease is the autosomal dominant mutation [20]. The pathophysiology underlying CNS diseases involve neuroinflammation, accumulation of distorted protein aggregates [24], and neuronal death [25].

The CNS and related diseases represent a serious health burden globally [26]. The therapy to treat CNS disorders is mainly hampered by the blood-brain barrier (BBB). BBB is a highly organized arrangement of the vasculature that has evolved naturally to restrict the entrance of molecules into the brain and to prevent the brain from potentially harmful microbes, to prevent microbials entering the bloodstream. Following these restrictions, most known therapies face the same hindrance from the BBB in entering the CNS and targeting the site of infection [27], whereas the BBB slowly allows the passage of nutrients and other related molecules with a smaller size which are a necessary function of the CNS [28].

Despite enduring efforts to deliver medicinal drugs to the brain tissue, these treatment regimens are compromised by low bioavailability due to the blood-brain barrier (BBB), a natural protective layer consisting of capillary endothelial cells, pericytes, and tight junctions [29]. This near-impermeable barrier impedes the entry of most macromolecules and allows only the minutest particles (<400 Da) to cross into the nerve tissue. Indeed, less than 5% of conventional therapeutic molecules in various stages of pharmaceutical development may penetrate this physiological barrier [30]. Therefore, CNS-targeted natural product formulations in nanocarriers hold infinite promise. Evidence suggests that nutraceuticals and phytochemicals exert therapeutic effects in neurological diseases, owing to their antioxidant, anti-inflammatory, and neuroprotective mechanisms [31,32]. Coupled with nanoscale delivery systems which improve solubility, enhance retention rates [33], and with the ability to permeate through the BBB (Figure 1), there is hope yet for effective treatment of neurological disorders. The following topic discusses two herbal compounds, curcumin and ginseng, and their significant potential for use in neurotherapeutics through nano-encapsulation.

A derivative of the South Asian turmeric plant rhizome, *Curcuma longa* (turmeric), the polyphenol curcumin has amassed quite a reputation in nutraceutical development for various diseases, including cancer and inflammatory conditions [34]. The healing properties of this yellow spice date back to ancient civilizations, featuring strongly in Ayurvedic therapy. Nevertheless, the relatively poor bioavailability due to dismal water solubility and rapid intestinal clearance impedes the therapeutic effects of conventional drug delivery, paving the way for the advent of nano-formulations.

The role of nanoparticulate systems to treat glioblastoma was recently highlighted using different strategies, such as improving their diffusion through the BBB [35]. In a glioblastoma study, Schmitt et al. demonstrated that curcumin encased in liposomal carriers (LipoCur) proved to be superior in reducing the proliferation and reactivity of human microglia and astrocytes (human fetal astrocyte cell line, SVGA) compared to the free compound. Immunostaining of murine organotypic brain segments challenged with lipopolysaccharide (LPS) for eight days, revealed that LipoCur was equally effective in reducing glial scarring [34].

Research on neuroblastoma models also corroborated that the anticancer properties of curcumin were significantly enhanced through nanocarrier delivery [36]. Curcumin nanosuspensions, consisting of stable nanoscale droplets of the compound with surfactants and biopolymers, improved solubilization and increased intravenous plasma distribution to vital organs compared with standard curcumin. Higher levels of curcumin in male Wistar rat brains were discovered when delivered via Tween 80-coated nanoparticles (NPs) in comparison to curcumin solution [37]. The mechanism of action exerted by curcumin in neuroblastoma cells involved the regulation of PTEN–Akt (phosphatase and tensin homolog-protein kinase B), NF-κB (nuclear factor kappa-light-chain-enhancer of activated B cells), and p53 (tumor protein P53), signaling, activation of the apoptosis cascade, and mitochondrial dysfunction [36].

In another study, dextran-coated cerium oxide nanoparticles (CNP-Cur), containing curcumin, induced significant cell death in an in vitro model of neuroblastoma. The formulation enhanced oxidative stress and triggered caspase-dependent apoptosis targeted to MYCN-amplified tumor cells and sparing non-MYCN augmented populations [38]. The synthesis of nanoparticle shells themselves could use natural compounds, such as silk fibroin of the *Bombyx mori* silkworm. The biocompatible and biodegradable properties add to the allure of the curcumin-loaded silk fibroin NP formulation, which conferred enhanced and tumor-specific cytotoxicity in neuroblastoma cells [39].

Aside from pure curcumin, combinations of herbal compounds that exhibit synergistic effects were also a subject of interest. A 10–25 µg/mL curcumin–piperine combination loaded into zein-chitosan nanoparticles (CPZChN) successfully reduced SH-SY5Y neuroblastoma cell viability by half, with encapsulation efficiencies of 89% (curcumin) and 87% (piperine) respectively [40]. Multi-compound bioactive complexes like curcumin–piperine are challenging to create, due to their distinct polarities. To overcome this, the two components were encased in core-shell NPs through antisolvent precipitation and progressive layering of zein-hyaluronic acid, followed by chitosan coating [41].

Aside from the CNS cancers, chronic degenerative conditions such as Alzheimer’s disease (AD) fall under intense scrutiny from researchers. Characterized by neurofibrillary tangles of tau proteins and “senile”amyloid-β (Aβ) plaques in the brain, the condition is notoriously challenging to diagnose and treat due to the late onset of symptoms, usually decades after the onset of pathological changes [42]. Neuro-inflammation is recognized as one of the primary mechanisms underlying neuronal degeneration, and thus is a target for natural product (NP) research. Malvajerd and colleagues encapsulated curcumin in solid lipid nanoparticles (SLNs) (entrapment efficiency 82 ± 0.49%) and nanostructured lipid carriers (NLCs) (94 ± 0.74%), and found that NLCs resulted in the highest bioaccumulation of curcumin in rat brains compared to free compound and SLNs [43].

A variant of lipid NPs, lipid-core nanocapsules (LNC), were used as curcumin carriers in an AD model of aged mice injected with β-amyloid 1–42 peptide. Significant behavioral changes indicating reversal of degeneration and reduced inflammatory cytokine levels were observed in the mice which received the loaded LNCs [44]. Recently, Nakama and colleagues proposed an analytical method to quantify curcumin and meloxicam in an LNC co-nanoencapsulation trial. They validated the encapsulation using a HPLC-DAD technique, which has been proven non-toxic to male Swiss mice [45].

Similar models, using curcumin-loaded SLNs (SCLNs) of about 86 nm in diameter, reduced LPS-induced neuroinflammation in BV-2 microglial cells by hindering nitric oxide (NO) production. Proinflammatory cytokines, including TNF (tumor necrosis factor)-α, IL (interleukin)-1β, and IL-6, were also more strongly inhibited compared to free curcumin [46]. Both in vitro and in vivo year-old 5xFAD mice models for SCLN delivery were evaluated by Maiti and colleagues, who reported enhanced curcumin penetration and neuroprotection. The assessment of Aβ plaque load and pre-frontal cortex (PFC) and hippocampus neuronal morphology revealed a down-regulation of pyknotic neurons and anti-inflammatory effect conferred by the curcumin NPs [47].

Poly (d,l-lactic acid-co-glycolic acid, PLGA) and polymer NPs are known to be biocompatible and thus show great potential in disarming neuroinflammation in another 5xFAD mice Alzheimer’s disease model [48]. Huang and team also achieved positive outcomes using novel PLGA NPs added with a BBB-penetrating (cyclic CRTIGPSVC) peptide, and co-administering curcumin with Aβ generation inhibitor. Transgenic AD mice responded well to treatment, with improved spatial memory scores and enhanced ’new-object recognition’ ability [49]. Co-administration with phytol and selenium in PLGA NPs have also been proven to inhibit β amyloid aggregation in various AD models [50,51].

Venturing into intranasal delivery, Zhang et al. compared two different formulations, namely CUR-encapsulated chitosan-coated poly (lactic-co-glycolic acid) nanoparticles (CUR-CS-PLGA-NPs) and hydroxypropyl-β-cyclodextrin-encapsulated CUR (CUR/HPβ-CD inclusion) complexes. Both showed anti-inflammatory potential in BV-2 cells and higher bioavailability in vivo, although the latter was evidently more effective by 1.12 fold (plasma)and 2.57 fold (brain) [52]. Other NP formulations include curcumin-conjugated superparamagnetic iron oxide (SPIO) particles < 100 nm in diameter, resulting in biocompatible curcumin magnetic nanoparticles (Cur-MNPs). Administration of Cur-MNPs to Tg2576 mouse brains revealed co-localization of the particles with amyloid plaques, enabling less invasive diagnosis of AD using MRI (magnetic resonance imaging) [53]. Clearly, curcumin-encapsulated NPs have captivated the pharmaceutical drug development sector for neurotheranostics and advancing to the next stage of clinical trials would be warranted.

Another ’superherb’, with its medicinal use dating back to ancient Chinese and Korean civilizations, the ginseng plant (*Panax ginseng* C. A. Meyer) is traditionally consumed to enhance alertness, cognition, memory, and overall well-being. Its purported effects on the CNS form the basis of neurotherapeutic drug development from numerous ginseng-derived bioactive compounds such as ginsenoside, gintonin and Compound K. These phytochemicals have been evaluated for curative and preventive properties in neurodegenerative diseases, including AD, PD, HD, and even depression [54,55]. Mechanistic studies suggest anti--apoptotic, antioxidant, neurogenesis, and inhibition of amyloid beta aggregation [56] in AD studies. A detailed review of bioactive components of *P*. *ginseng* and their effects on AD pathogenesis was recently reported by Razgonova and colleagues [57].

Aalinkeel and team investigated the neuroprotective effect of Ginsenoside Rg3 combined with Thioflavin T, an Aβ diagnostic, encased in biodegradable PLGA NPs. An in vitro model was used to evaluate the efficiency of BBB penetration and underlying mechanistic pathways of bioactivity. They concluded that PLGA-Rg3 NPs may prove to be a potent nanodelivery tool for the treatment of neurological disease [58]. Ginsenoside was also evaluated for anti-PD therapy through its antioxidant effect, modulation of glutathione level, ROS-mediated NF-κB signaling, iron transport, and subsequently reduced iron accumulation in the substantia nigra [31].

Other groups evaluated the bioactivity of Compound K (CK), a metabolite from the biotransformation of ginsenosides Rb1, Rb2, and Rc. Other well-studied ginsenosides include Rd, Re, and Rg131. As the most bioavailable component of ginsenoside metabolism, its neuroprotective and cognition-enhancing effects have been characterized in animal models of AD [59]. Although CK is regarded as safe and well-tolerated, findings from clinical trials are marginal and based on CK-rich fermented red ginseng. Delivery of CK through nanocarriers was reported to override its limitations of efflux, poor water solubility, and membrane permeability, which impeded the research progress into clinical trials [60,61].

Kim et al. established a formulation of red ginseng water extract with gold nanoparticles (WERGGN) and established its antioxidant activity through DPPH, ORAC and ABTS assays. WERGGN treatment on neuron-like PC-12 cells revealed cytoprotective effects, mainly due to the decreased intracellular oxidative stress. In addition, levels of neurotransmitter degradation enzymes such as acetylcholinesterase and butyrylcholinesterase were also inhibited, suggesting that WERGGN promoted synaptic impulse transmission [62]. Nonetheless, the dosage of ginseng extract, when co-administered with a synthetic drug (e.g. selegiline), determines the pharmacokinetics in the body: lower doses result in poorer bioavailability due to CYP1A2 induction, while higher concentrations cause inhibition of CYP3A4 and thus enhanced systemic exposure [63].

Although the root of *Panax ginseng* is generally the primary source of extracted bioactive compounds, its leaves may be equally valuable. Singh et al. characterized and validated gold- and silver-NPs with fresh ginseng leaf extract, demonstrating significant antioxidant and anti-inflammatory activity, and anti-carcinogenic effects or biocompatibility in several tumorigenic or normal human cell lines, respectively [64]. Studies on the anti-carcinogenic effects of polymer–ginsenoside nanoconjugates revealed improved bioavailability into the tumor sites, efficient drug release, and oncogene-targeting mechanisms [65]. In a glioma spheroid model, Zhu et al. developed a multifunctional ginsenoside Rg3-based liposomal system (Rg3-LPs) which demonstrated a superior anti-proliferation effect on C6 glioma cells compared to cholesterol liposomes (C-LPs) when embedded with paclitaxel (PTX). In C6-bearing mice//rats, Rg3-PTX-LPs triggered expansion of CD8+T-cell populations, thus enhancing cancer-ridding immune surveillance. The researchers surmised that ginsenoside Rg3 shows a synergistic effect with synthetic chemotherapy drugs and provides an effective delivery tool for neurological tumors [66]. In another study, a nano-ginseng formulation, ’ginsenoside Rb1/protopanaxadiol nanoparticles’ (Rb//PPD NPs) of ~110 nm in size, showed increased drug-loading efficiency (~96.8%) and capacity ((~27.9 wt%), although this was targeted on the Lewis lung cancer (LLC) cell line and xenograft [67].

**Figure 2:**
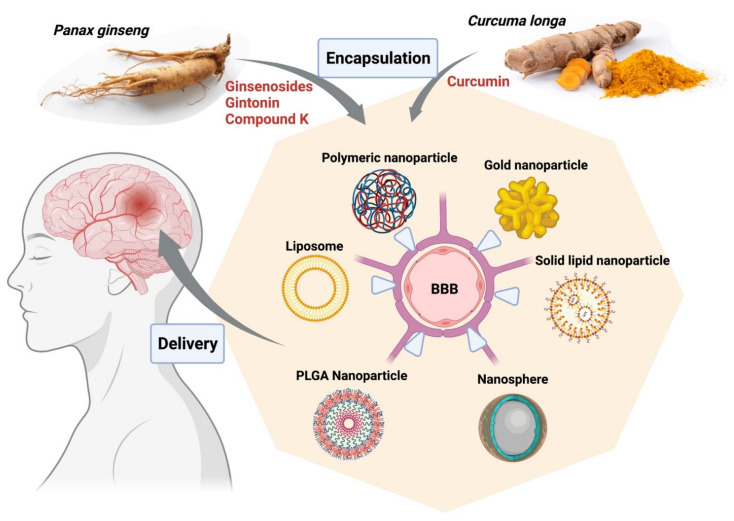
Encapsulation of bioactive compounds from herbal extracts of *Curcuma longa* and *Panax ginseng* into various nanoparticle platforms facilitates delivery to targeted areas in the brain from the blood, improves solubility, and enhances retention rates. The figure was made with www.biorender.com (access date: 12 January 2022). Abbreviations: BBB, blood-brain barrier; PLGA, poly (d,l-lactic acid-co-glycolic acid).

In a nutshell, derivatives of *Panax ginseng* display great potential in the treatment of neurodegenerative conditions as well as various cancers, including gliomas, and should be explored further (Figure 2). Nano-formulations of active compounds or extracts from curcumin and ginseng for neurological disorders described in this topic are briefly listed in Table 1.

It is essential to note that nanomaterials have potential limitations and the adverse effects on CNS were described previously [69,70]. For example, one study showed that female mice that were intranasally treated with titanium dioxide nanoparticles (TiO_2_ NPs) (0.5 mg daily for 1 month) produced apoptosis, affected brain development, and oxidative stress (caused increased lipid peroxidation, protein oxidation, catalase expressions, and release of glutamic acid and nitric oxide) [70]. These authors also concluded that an intranasal spray of TiO_2_ NPs could be translocated into the CNS and cause potential lesions of the brain [70]. Similarly, Fe_2_O_3_ magnetic NPs (0.15–15 mM) also showed toxicity and c viability in a neuronal cell line in vitro [71]. Whereas another study showed that toxicity of zinc and silicon NPs is relatively low, but the neuronal toxicity of NPs depends on the materials used [69,72,73]. Therefore, the toxicity of NPs is present, and depends on the formulation, particle size and concentration or treatment protocols of specific NPs.

### 3.2. Nano-Phytopharmaceuticals in Cardiovascular Disorders

Cardiovascular disorders are linked to the heart and blood vessels, implicating coronary heart diseases (e.g., heart attack), cerebrovascular disease (e.g., stroke), elevated blood pressure (e.g., hypertension), peripheral artery disorders, rheumatic heart disease, congenital heart disease, and heart failure [74]. A large number of individuals die of cardiovascular diseases yearly compared to other diseases around the globe [74]. The risk factors associated with cardiovascular disorders mainly include tobacco use, unhealthy diet and obesity, lack of physical activity, inappropriate use of alcohol, hypertension, diabetes, and hyperlipidaemia [75]. Cardiovascular disorder refers to a condition that affects the heart or blood vessels mostly associated with deposits of fats in the arteries, a condition specifically known as atherosclerosis that can lead to blood clots. Most often, CVD is associated with damage to the arteries, not only of the heart, but also of other major organs, such as kidneys, brain, and eyes [76]. It is one of the leading causes of death worldwide. Noticeably, some pressing concerns are related to rising heart disease mortality rates among younger adults [77] A wide range of complications may emerge within cardiovascular systems, such as rheumatic heart disease, endocarditis, and abnormal conduction system [78]. There are many different types of CVD, but the main four are (i) Coronary heart disease (CHD) which occurs when the flow of oxygenated blood to the heart muscle is reduced or totally blocked. This condition leads to angina, heart attack, and heart failure; (ii) Stroke and transient ischemic attack (TIA), both conditions block the blood supply to the brain, but the former can cause brain damage and possibly death, while the latter brings about temporary interruption of blood flow to the brain, hence it is called a ’mini-stroke’; (iii) Peripheral arterial disease happens when the blockage of arteries involves the limbs, often the legs; (iv) Aortic disease involves the biggest artery, the aorta. This artery weakens and bulges outwards, and may be life-threatening due to its possible bursting that could lead to profuse bleeding [77,79].

Cardiovascular disorders, associated with many factors, involve various congenital and acquired ailments. Cardiovascular disorders include atherosclerosis with its subtypes, such as coronary, cerebral, and peripheral artery disease, along with two main complexities, myocardial infarction and ischemic stroke, heart failure, cardiac valvulopathies and arrhythmias, rheumatic heart disease, congenital heart disease, deep vein thrombosis with its specific complexities, and pulmonary embolism [80]. It comprises the main noncommunicable reason for death in Europe specifically, and also globally [80].

CVD requires immediate attention and treatment to prevent further damage to avoid harmful clots from forming in blood vessels and plaque build-up [81]. Medications are any one or combinations of the following: anti-coagulants, anti-platelet agents, dual anti-platelet therapy, angiotensin-converting enzyme (ACE) Inhibitors, angiotensin II receptor blockers, angiotensin receptor-neprilysin inhibitors, beta blockers, calcium channel blockers, cholesterol--lowering medications, digitalis preparations, diuretics, and vasodilators [81,82]. Each type of medication has a specific action to prevent the formation of a blood clot that could cause a blockage in the blood vessel. In worst conditions, patients may undergo medical procedures such as coronary angioplasty and stent implantation, thrombolytic therapy, coronary artery bypass graft surgery (CABG), artificial pacemaker surgery, defibrillation, and heart valve surgery [82] 

Since CVD require most often long-term medication, treatment regimens become complex and create a burden for the patient especially when multiple medicines are prescribed and must be taken for life [83,84] Although these therapeutic drugs have been successful in halting the progression of the disease, thereby improving the quality of life of patients, most of these cure only the symptoms and may not repair or regenerate the damaged tissues. In addition, CVD medication has different side effects, such as antiplatelet drugs may cause diarrhea, rash, or itching [83]. Others can cause abdominal pain, headache, chest pain, muscle aches, and dizziness. In the case of anticoagulants, their side effects can lead to bleeding and necrotic or gangrenous skin. Given the excessive side effects of current therapies, alternative therapeutic approaches like medicinal plants and natural products are preferred. Against this premise, a better treatment for CVD that would not burden the patients is necessary. Hence, it is important to explore new technologies and drugs to lessen the use of conventional treatments. The lower toxicity, chemical diversity, cost-effectiveness, and therapeutic potentials of natural products make them the popular choice of medicine compared to other products [85]. With the combination of nanoformulation methods to deliver phytomedicines, it becomes more effective with improved solubility, bioavailability, circulation time, surface area-to-volume ratio, nil systemic adverse side effects, and drug delivery efficiency of these medications. The introduction of nanomedicine using a combined nanotechnology with drugs or diagnostic molecules has improved the ability to target specific cells or tissues that require treatment and repair. These nanomaterials are produced on a nanoscale level and are safe to introduce into the body. Hence, applications for nanotechnology in medicine include imaging, diagnosis, or the delivery of drugs that will help medical professionals treat various diseases including cardiovascular diseases [86,87]. The functionality of the most recent nano-formulated medicinal plants and/or natural products against various cardiovascular conditions such as hypertension, atherosclerosis, thrombosis, and myocardial infarction is expected to be maximized.

Some plant extracts/compounds used for treatment of CVD that are nano-formulated for efficient delivery to their target are listed in Table 2. Among the extracts used for CVD, curcumin, quercetin, and resveratrol were the most applied natural products, respectively. However, curcumin, despite its curative potential, has poor aqueous solubility and consequently, minimal systemic bioavailability along with rapid degradation [88]. These characteristics restrict the utilization of curcumin at medical perspective. Liposomes have found uses in drug delivery of poorly water-soluble drugs. Such drug-loaded liposomes can be fabricated by a wide variety of nanotechnology methods such as ethanol injection, thin-film hydration, sonication, high-pressure extrusion, reverse-phase evaporation, calcium-induced fusion, and supercritical fluid methods, among others [88].

Quercetin acts as an antioxidant on cells but is hindered by its high metabolism rate. In order to control this, quercetin is encapsulated in poly [lactic-co-glycolic] acid (PLGA) nanoparticles [89]. This guarantees secure and controlled release of the quercetin, and permits cell enlistment, attachment, expansion, and articulation of heart proteins in local myocardium.

Resveratrol’s capacity for protective action makes it one of the commonly used compounds against CVD. However, its poor pharmacokinetic properties, such as low aqueous solubility, low photostability and extensive first-pass metabolism, result in poor bioavailability and hinder its clinical potential [90,91]. To resolve this issue, the use of lipid nanoparticles offer the possibility to develop new therapeutics. Further, solid lipid nanoparticles hold great promise for reaching the goal of controlled and site-specific drug delivery [85].

**Figure 3:**
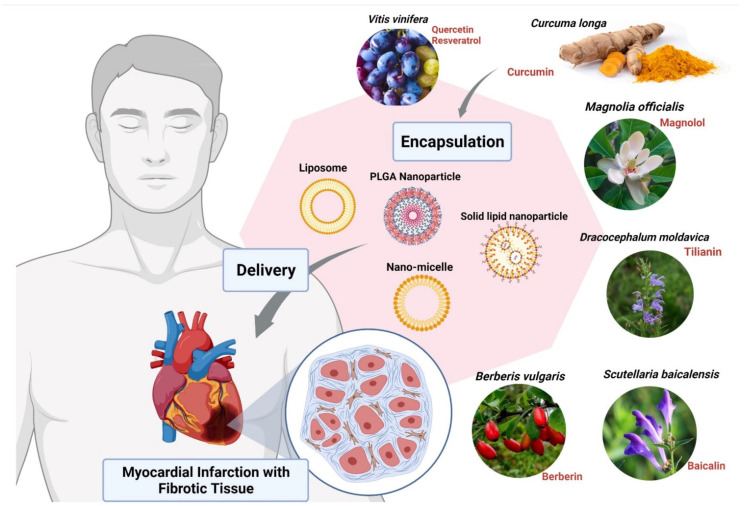
Encapsulation of bioactive compounds with nanoparticles from various herbal extracts to prevent cardiovascular disorders. Abbreviation: PLGA, poly (d,l-lactic acid-co-glycolic acid). The figure was made with www.biorender.com (access date: 12 January 2022).

Other compounds used for CVDs are Magnolols, Berberine, Tilianin and Baicalin. These compounds require nanoformulation to maximize their use. Magnolols as a phenolic polyhydroxy compound have poor aqueous solubility and low oral bioavailability which limit their clinical use. Therefore, various formulations such as liposomes [92], solid dispersions [93], emulsions [94], and nanoparticles [95] have been developed to ameliorate the water solubility and bioavailability of it (Figure 3). On the other hand, Berberine (BBR) has low bioavailability and also shows poor absorption through the gut wall (<5%) and bowel glycoprotein appears to contribute to its poor absorption, actively expelling the alkaloid from the lumen mucosal cells, hence the use of liposomes as nanoformulation works best for BBR [85,96]. Studies have shown that Tilianin eases ischemia-reperfusion-induced cardiomyocyte injury. However, its full effectiveness is prevented because of its insolubility in water. The use of nanoformulation based on Tilianin nano-micelles overcomes this obstacle, as does the polyethylene glycol compound, covalently attached to propylene sulfide-formed amphiphilic diblock polymers [97]. In the aqueous solution, Tilianin is encapsulated in a hydrophobic shell to form nano-micelles [85,97]. The Ph-PPS-PEG self-assembles into nanoscale micelles with a size of approximately 70 nm, termed “Tilianin-loaded micelles” (TLMs).

NPs have shown some adverse effects on cardiovascular systems previously. Mice exposed to zinc NPs in the stomach (5 mg/kg body weight) experienced cardiac impairment, increased lactate dehydrogenase, creatinine kinase and aspartate aminotransferase, which was lower in zinc microparticles at the same dose [98,99]. Therefore, cardiac toxicity of zinc nanomaterials may be associated with particle sizes as per Wang and colleagues [99].

The rapid elimination half-life in plasma and poor water solubility of Baicalin (BN) limits its clinical efficacy. Aside from its short half-life and low water solubility, its bioavailability is low [100]. In order to enhance its clinical efficacy, the Novel Baicalin-loaded PEGylated nanostructured lipid carriers (BN-PEG-NLC) have been used to improve the bioavailability of BN, to prolong retention time in vivo and to enhance its protective effect [85,101,102]. These nanotech applications aim to elude problems related to conventional drug delivery and possible negative systemic side effects. Once the nano-drug carriers have migrated locally, they provide the therapeutic payload delivery. This process eventually achieves therapeutic efficacy for a variety of cardiac disease indications [89].

### 3.3. Nano-Phytopharmaceuticals in Pulmonary Disorders

Globally, at least two billion people get infections of the respiratory system, which causes disability, death and imposes an enormous health burden [108]. An estimated 65 million people have moderate to severe Chronic Obstructive Pulmonary Disease (COPD), which cause three million deaths every year [109]. About 334 million people suffer from asthma [110,111]. Acute lower respiratory tract infections are among the top three causes of death and disability. Influenza alone kills approximately 650,000 people annually [112]. Pulmonary disorders are characterized as progressive and incurable diseases, that comprise one of the five leading reasons for death around the world. The risk factors involved in the disease are smoking, permanent bronchoconstriction, alveolar destruction, and airway and pulmonary vascular remodeling [113].

Obstructive Lung Disease is characterized as airflow disturbances with consistent respiratory ailments. Patients having obstructive lung diseases present breathlessness, sputum production, and intolerance to exercise. Dyspnea may be considered a symptom of the early stages of the disease, whereas heart failure can be the symptom of severe and chronic disease [114]. In addition to chronic bronchitis and lung emphysema, it also affects various systems in the human body [115].

Tuberculosis, another respiratory disease, is one of the 10 leading causes of death from a single infectious agent. In 2019, around 1.4 million people died from tuberculosis (TB), and an estimated 10 million people became ill. Multidrug-resistant TB (MDR-TB) remains a public health crisis and a threat to security health [116]. In addition, more than 100 million people suffer from sleep-disordered breathing and millions live with pulmonary hypertension. More than 50 million people struggle with occupational lung diseases. The reason for pulmonary infections is the potential widespread pathogens in our environment and infections acquired from other infected individuals [117]. Nosocomial transmission primarily contributes to the pulmonary infections of multidrug-resistant (MDR) bacteria like cystic fibrosis [118] and bronchiectasis [119]. Intensive care units [120] are frequent sources of recurrent cross infection. The common nosocomial pneumonia with pathogens are *Legionella*, [121], *Aspergillus* [122] and mucormycosis [123].

Pathogens causing respiratory infections are either well adapted to the human body or cause little or no disease in animals. In addition, some pathogens, such as *Mycobacterium tuberculosis* and viruses producing IL-10, change their genetic system to evade the host immune system [124]. Another challenge of the respiratory infections is the development of drug-resistant pathogens, for example, pan-resistant *Pseudomonas aeruginosa* [125] and extensively drug-resistant *M*. *tuberculosis* [126]. Some new developments in drug-resistant pathogens, such as methicillin-resistant *Staphylococcus aureus*, are new threats in some regions [127]. Viruses are also developing resistance against many antiviral agents, for example, the neuraminidase inhibitor-resistant influenza virus [128]. The constant modification of viral genomes results in the antigenic drift and antigenic shift responsible for the development of resistant strains. New pathogens, like SARS, MERS and SARS-CoV-2, are serious threats to the world population [129]. The continuous use of antibiotics is creating a new niche for other pathogens, for example, bronchiectasis and cystic fibrosis, and non-tuberculous mycobacteria [130].

To curb the increasing threat of respiratory diseases, there is an immediate need to explore some alternatives to the use of antibiotics, and an ecofriendly, cost effective, efficacious and durable strategy to combat these global respiratory problems. Thus, the field of nanotechnology provides a promising technology against respiratory diseases. In the past, various studies have shown very effective and interesting results. In addition to their antiviral properties, the application of nanoparticles (NPs) provides a potential strategy to manage infections caused by multidrug-resistant organisms (MDROs) [131,132,133]. NPs exhibiting antibacterial activities can target multiple biomolecules and have the potential to reduce or eliminate the evolution of MDROs [134]. Plant extracts are complex mixtures that provide a rich arsenal of molecules, such as flavanones, flavones, flavonols and chalcones, fatty acids, amino acids, terpenoids, aldehydes, and alcohols [135], with high redox potential [136]. Furthermore, biogenic synthesis produces potential, stable, and better-defined materials [137,138].

Silver nanoparticles (AgNPs) have shown broad applications in the medical system, such as anti-inflammatory, anti-angiogenesis, antiplatelet, antifungal, anticancer, and antibacterial activities [139,140,141,142]. Nowadays, AgNPs have been reported as biomedical therapeutic agents, such as wound dressings and long-term burn care products and antibacterial lotions [143]; They also exhibit antiviral activities against influenza A virus, hepatitis B virus, human parainfluenza virus, herpes simplex virus, and human immunodeficiency virus [144,145]. They prevent the anchoring and binding of the virus to the host and cell receptor, respectively, and hence they deactivate the virus by denaturing the surface.

AgNPs inhibit the binding of the virus by interacting with the glycoprotein (gp120) of the sulphur-bearing groups distributed in the lipid membrane of the virus [146]. In another study, the virus inactivation was observed when the nano-silver was combined with the nucleic acid of the virus and modified the structure of capsid and affected the replication [147]. Along with such surface modification, AgNPs also show synergistic antiviral activity. In one study, it was reported that curcumin, when used as both a reducing and stabilizing agent prevented viral replication, and blocked the budding of viruses [148]. Similarly, the AgNP surface, when modified through chemical methods with drugs like zanamivir, amantadine and oseltamivir, was able to interact with virus particles directly, which destroyed the virus and blocked its entry [149].

**Figure 4:**
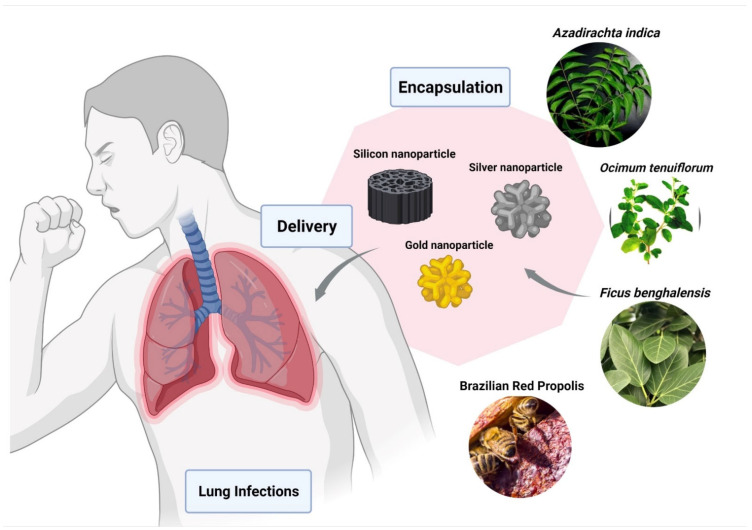
Encapsulation of bio-active compounds from various herbal extracts with silver, silicon, or gold nanoparticles to improve delivery in lungs and to prevent pulmonary disorders. The figure was made with www.biorender.com (access date: 12 January 2022).

Du et al. showed the Ag2S NCs has excellent antiviral and immunomodulating activities against the porcine epidemic diarrhea virus (PEDV) [150]. Ag2S NCs also showed a comparable inhibitory effect of the virus on other RNA viruses, such as porcine reproductive and respiratory syndrome virus (PRRSV). Various reports on the use of natural products as reducing and capping agents for nanomaterial synthesis has been well documented [151,152]. Leaves of plants like *Azadirachta indica* [153], *Ocimum tenuiforum* [154], and *Ficus benghalensis* [155] have been used for the synthesis of AgNPs (Figure 4). The medicinal properties and broad-spectrum antimicrobial activities of Tulsi leaves were known for over a thousand years [156].

Gold nanoparticles (AuNPs) provide superior properties [157], and various methods for the preparation of gold nanoparticles have been well reported [139]. Compared to silver nanoparticles, AuNPs display better results in in vivo studies. AuNPs can interact with the haemagglutinin (HA) glycoprotein and can oxidize the disulfide bond, resulting in the inactivation of the virus [158]. Surface properties are useful in targeting the virus. The sulphonates are organic sulphates that were used to interact with the capsid proteins of the virus cell and prevent the HA activity [159]. In one study, it is reported that Brazilian Red Propolis (BRP), a product of bees, exhibits anti-inflammatory, anti-tumor, antioxidant, and antimicrobial activities [160]. It described the biosynthesis of AuNPs using BRP extract (AuNPextract) and its fractions (AuNPhexane, AuNPdichloromethane, AuNPethyl acetate) and evaluated their structural properties and their potential against micro-organisms and cancer cells.

Many drugs failed to treat viral infections, mainly due to drug release efficiency. To resolve this issue, mesoporous silicon nanoparticles are used as antiviral drug delivery carriers [161]. Compared to silver and gold nanoparticles, silicon nanomaterials are less cytotoxic and more biocompatible. The glycosaminoglycan (GAGs) mimetic-functionalized solid and mesoporous silica nanoparticles have shown antiviral activities that inhibit the entry of herpes simplex virus (HSV) type 1 and type 2 viruses into host cells [162].

In nanoscience, the carbon dots (CDs) also play an important role due to their unique properties. CDs prevent the viral infection as they have hydroxyl and carboxyl groups on their surfaces that interact with viral membranes [163]. To improve the activities of CDs, antiviral agents like plant extracts are grafted on the surface that involves a two-step reaction. The functionalized antiviral agent shows the inhibition of the virus into the cell that has a broad-spectrum action for the enveloped and the non-enveloped viruses [164].

Another important nanotechnology-based materials are based on graphene, (GO), which also have biomedical applications [165]. To prevent viral infection in the host cell, GO has been used with organic and metallic nanoparticles that show antiviral activities [166].

In one study, different nanocomposites of graphene oxide (GO) with Ag and iron oxide (IO) NPs were shown to exhibit both antimicrobial and magnetic properties. In another study, Ocsoy et al (2017) [167] reported that silver nanoparticles (Ag NPs)-decorated magnetic graphene oxide nanocomposites showed antimicrobial activities against *Staphylococcus aureus* (Gram+), *Escherichia coli* (Gram−) bacteria, and *Candida albicans* fungus.

Another material suitable for nanotechnology is selenium. In the in vivo study, selenium nanoparticles (SeNPs) showed antiviral efficiency [168]. Mainly, these nanoparticles depend on the quenching of free radicals in the host, and the prevention of the mitochondrial depolarization and apoptosis [168]. Zinc is another material that can be used in nanotechnology. In one study, ZnO-NPs, when inoculated with the virus, prevented cell adhesion from displaying the viral antigens to the T cells. The in vivo study showed that it has 90% antiviral activity and it mimics the couple cells [169]. In another study, ZnO-NPs and graphene carbon material showed antibacterial activities against Gram negative bacteria *Escherichia coli [E*. *coli]* and Gram-positive bacteria *Staphylococcus aureus* [170]. Graphene oxide-Ag nanoparticles formed from the extract of *Pistacia atlantica* leaves showed antimicrobial activities against *S*. *aureus, S*. *saprophyticus, S*. *pyrogenes* and *B*. *Subtilis* [171]. Calcium as a nanomaterial also has some unique features, and it can be used in nanotechnology. In one study, calcium phosphate nanoparticles (CaPNPs) showed antiviral activities [172]. The CaPNPs covalently modified with the alum and the two peptides generated efficient antibodies to block the cell from infection by the virus [173].

Self-assembling polypeptide nanoparticles that repetitively display a SARS B-cell epitope from the C-terminal heptad repeat of the virus’ spike protein represent a promising platform for vaccine design [174]. It has also been reported that novel nanoparticle vaccine containing full spike protein of MERS CoV and SARS-CoV resulted in higher-titer neutralizing antibody in vaccinated mice [175]. The vaccination in combination with an adjuvant, matrix M1, boosted the neutralizing antibody titer [176].

Nanoparticles also have shown toxicity in lungs and other parts of the respiratory system, as observed in several preclinical and in vitro studies. Carbon black NPs showed increased release of pro-inflammatory cytokines in the alveoli that could potentially induce migration of macrophages in a lung cell line (type II alveolar epithelial cells) (in vitro) [177]. Another study showed TiO_2_ NPs showed increased oxidative damage (increased nitric oxide and H_2_O_2_ levels), lipid peroxidation, and micronuclei formation in a human bronchial epithelial cell line [178]. Another study also reported that a daily dose of 40 mg/kg body weight of TiO_2_ NPs over a period of three days increased serum albumin, alkaline phosphatase (ALP), acid phosphatase (ACP) levels, and pulmonary toxicity in mice [179]. Importantly, pH of the formulation, particle size, surface area, and aggregation of particles may play an important role in the pulmonary toxicity of TiO_2_ NPs [179].

### 3.4. Nano-Phytopharmaceuticals in Gastro-Intestinal Disorders

Gastrointestinal disorders are recognized as an important health issue because of increasing number of individuals diagnosed with colorectal cancer, coeliac disease, and inflammatory bowel disease. Early diagnosis plays a vital role in survival, especially in colon cancer, which can be achieved with endoscopy or colonoscopy [180]. The gastrointestinal tract consists of a dynamic linkage where several layers frame a coordinated functional unit as a semipermeable ecosystem with multiple layers [181]. The association between host and gut microbiota is regulated by a mutual and complex symbiosis [182]. This association is continuously being tested with various factors, which may result in the destruction of the microbial community [182]. Dysbiosis, a disturbance in the composition of microbiota, has been related with severe complications of the gut, for example, in the pathogenesis of inflammatory bowel disease, irritable bowel syndrome and other GI disorders [183,184]. A dysregulated immune response to environmental factors leads to inflammatory disorders of the digestive system, such as inflammatory bowel syndrome, including ulcerative colitis or Crohn’s disease [185]. Irritable bowel syndrome comprises the most recognized condition. In addition to other contributing factors, dysbiosis contributes as a major factor [186]. Similarly, diverticular disease is responsible for a significant burden in Western and developed nations, socially and economically. The pathophysiology of diverticular disease is not exactly known, though less intense inflammation and diversification of gut microbiota are considered to be the main factors associated with the disease. Gut microbiota specifically play a role in diverticula progression and in basic diverticular disease, which is defined as a disease with mild symptoms, for example pain in the abdomen or variations in bowel habits [182,183].

There are numerous gastrointestinal diseases that can occur in different areas of the gastrointestinal tract [(GIT)], from the mouth to the anus [187]. Although the mouth is part of the GIT, mouth-related diseases are rarely considered as part of gastrointestinal diseases. The most common types of GIT-related diseases are Crohn’s disease, ulcerative colitis, irritable bowel syndrome, and colorectal cancer. Their common symptoms include diarrhea, abdominal pain, stomach bloating, and gastrointestinal bleeding [188]. Apart from lifestyle and genetic factors, infectious agents such as parasites, virus, and bacteria are also known as the etiological agents for most of the gastrointestinal disorders. Interestingly, most gastrointestinal disorders can be cured with traditional or modern medicine. Phytotherapy based on herbal medicines has been one of the important resources to treat gastrointestinal disorders for many years. Their efficacies could be improved by using nanotechnology with lesser side effects.

**Figure 5:**
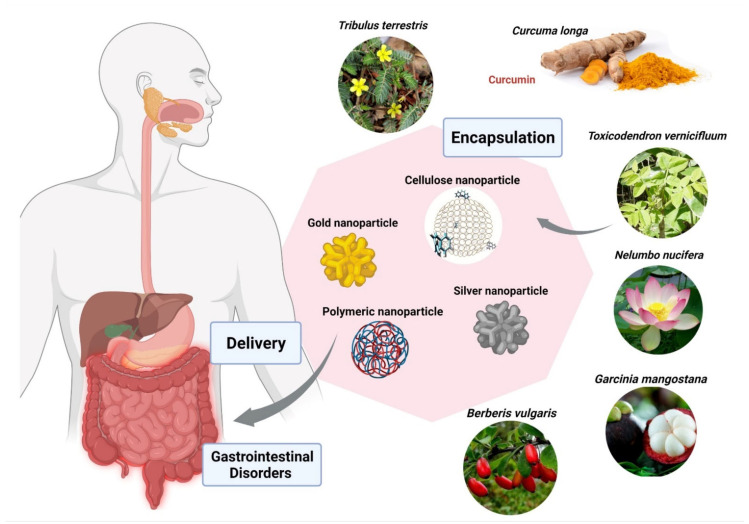
Encapsulation of bioactive compounds from various herbal extracts with silver, gold, cellulose, and polymeric nanoparticles to improve delivery in the gastrointestinal tract, and to prevent or cure various diseases. The figure was made with www.biorender.com (access date: 12 January 2022).

*Berberis vulgaris* and *Curcuma longa* extracts encapsulated in cationic polymer EPO (Figure 5), demonstrated significant anti-parasitic activity against *Entamoeba histolytica*. Furthermore, *B*. *vulgaris* encapsulated within EPO showed IC_50_ of 26 ppm in comparison with the free extract having IC_50_ of 34 µg/mL. The IC_50_ of *Curcuma longa* loaded within EPO was found to be 19 ppm in comparison with the free extract having IC_50_ of 38 µg/mL [187]. Another group of researchers used cerium oxide nanoparticles encapsulated with *Nelumbo nucifera* flower extract against the human colon cancer cell line (HCT 116), exhibited IC_50_ of 4.16 mg/mL [187]. *Garcinia mangostana* has been widely used for medical applications. *G*. *mangostana* extract loaded within ethylcellulose and methylcellulose nanoparticles proved a highly protective agent for stomach ulcers with a MIC value of 6.25 ug/mL against *Helicobacter pylori*, which is almost the same as metronidazole. A study done by Saravanakumar and team found that silver nanoparticles encapsulated with *Toxicodendron vernicifluum* extract were effective against enteropathogenic bacteria, *E*. *coli* and *H*. *Pylori,* with MIC 8.12 µg/mL and 18.14 µg/mL. *Acorus calamus* Lim extract loaded within silver nanoparticles has shown significant inhibition in the formation of *H*. *pylori* at a concentration of 350 µg/mL. Metallic nanoparticles demonstrated promising results in inhibition of enteropathogenic bacteria in various studies. Gold nanoparticles loaded with extracts of *Tribulus terrestris* showed effective activity against *H*. *pylori* in a size-dependent manner, with MIC of 16.75 µg/mL at 55 nm and MIC of 18 µg/mL at 7 nm size of gold nanoparticles, respectively [187](Figure 5; Table 3).

Apart from benefits, NPs showed adverse events, such as selenium and selenite nanoparticles in mice (2 or 4 mg/kg body weight daily) treated over a period of 15 days which showed more toxicity (growth suppression, increased liver toxicity, and reduced superoxide dismutase activity) in selenite NPs [189].

Nanotechnology is a vital tool for its applications in medical science, given that it is possible to obtain a variety of nanocarrier characteristics that include their constituents, size, shape, bioavailability, surface properties, and target specificity to achieve or enhance desirable pharmacological targets [187]. A number of strategies have been implemented to increase the drug-target specificity. Recently, several studies have reported the improved efficacy of herbal extracts for gastrointestinal disorders when associated with nanomaterials mainly due to oral absorption, greater stability, and simulated intestinal fluid [3,4]. Pure herbal medicines are often considered less effective in comparison due to their size and reduced intestinal absorption when administered orally. However, plant extracts loaded on nanoparticles are more stable in high protein environments and help to increase target specificity [4]. Solubility is always considered a major concern associated with plant extracts, which can be successfully upgraded using nanosystems [189]. These are the reasons behind a pharmacological loss associated with plant extracts and such problems can be overcome using novel drug delivery systems such as nanotechnology. The advantages of using these novel delivery systems include better absorption by smoothing diffusion through the epithelium, modification of pharmacokinetics, enhancement of intracellular penetration, and distribution with reduced toxicity, overcoming resistance and lowering cost. Nanoparticles are extremely stable in harsh conditions like sterilization temperatures [189].

## 4. Conclusions

The role of nanotechnology has been evident in the field of public health. This review article highlights the role of nanotechnology applied to the treatment of various diseases. The use of nanomedicines can play a significant and promising role in the successful application of plant-based medicine to treat various diseases and thus improve patient health outcomes. Nanoparticles and their use in novel drug delivery systems may provide solutions to the problems faced by conventional or allopathic medicine, as well as the limitations of plant-based medicine delivery. Despite the great advent of nanotechnology, as explained in this review, there are some limitations mentioned below that should be developed and clarified in future research. For example, the unbalanced distribution of the inorganic parts of nanostructures in the brain may induce adverse effects. Additionally, the concentration of these nanomaterials in the brain may cause neurotoxicity, given their interference with mitochondrial activity, autophagy, apoptosis, and neuronal inflammation. Thus, in vitro and preclinical studies have shown that, by combining nanomaterials with plant-based drugs, a more adequate delivery to target cells or tissues is possible, increasing their effectiveness. However, the low reproducibility of in vitro and in vivo effects of many herbal medicines prevents these medications from reaching the clinical trial phases, so it will be necessary to develop concerted actions through in vitro and in vivo protocols that can be comparable. Therefore, preclinical pharmacodynamic and pharmacokinetic studies of the phytoconstituents involved are also important in view of their efficacy and safety. In addition, studies focused on systemic toxicity are still required to know its adverse effects in order to promote its future application. Future research studies must address describing critical process parameters, techniques, challenges, and solutions for the development of nano-phytopharmaceuticals.

## Figures and Tables

**Table 1:** Nano-formulations of active compounds or extracts from curcumin and ginseng for neurological disorders.

Nanoformulation	Phyto-Pharmaceutical	Effect	References
Liposomal carriers	Curcumin (LipoCur)	Reduced proliferation and reactivity of human microglia and astrocytes; reduced glial scarring	[34]
	Ginsenoside Rg3 in combination with paclitaxel (Rg3-PTX-LPs)	Anti-proliferative effect on C6 glioma cells; triggered expansion of CD8 + T-cell populations in C6-bearing mice/rats	[66]
Tween 80-coated nanoparticles	Curcumin	Higher accumulation of curcumin in Wistar rats; improved solubilisation and plasma distribution to vital organs	[37]
Dextran-coated cerium oxide nanoparticles	Curcumin (CNP-Cur)	Enhanced cell death in neuroblastoma cells through caspase-dependent apoptosis	[38]
Silk fibroin nanoparticles	Curcumin	Enhanced tumour-specific toxicity in neuroblastoma cells	[39]
Zein–chitosan nanoparticles	Curcumin-piperine combination (CPZChN)	Reduced SH-SY5Y neuroblastoma cell viability by half	[40]
Solid lipid nanoparticles (SLNs);Nanostructured lipid carriers (NLCs)	Curcumin	Highest bioaccumulation of curcumin in rat brains compared to free compound and SLNs	[43]
		Reduced LPS-induced neuroinflammation in BV-2 microglial cells	[68]
		Downregulation of pyknotic neurons and anti-inflammatory effect	[47]
Lipid-core nanocapsules (LNC)	Curcumin	Reversal of degeneration and reduced inflammatory cytokine levels in AD mice model	[44]
Poly (D,L-lactic acid-co-glycolic acid, PLGA) polymer nanoparticles	Curcumin	Reduced neuroinflammation in 5xFAD mice	[48]
	Curcumin co-administered with phytol and selenium	Inhibited β amyloid aggregation in AD models	[50,51]
	Ginsenoside Rg3 combined with Thioflavin T	Effective penetration of an in vitro blood-brain barrier	[58]
Novel PLGA nanoparticles added with a BBB-penetrating (cyclic CRTIGPSVC) peptide	Curcumin with Aβ generation inhibitor	Transgenic Alzheimer’s disease mice had improved spatial memory scores and enhanced ’new-object’ recognition	[9]
Chitosan-coated poly (lactic-co-glycolic acid) nanoparticles (CUR-CS-PLGA-NPs); Hydroxypropyl-β-cyclodextrin-encapsulated CUR (CUR/HP-β-CD inclusion) complex	Curcumin	Anti-inflammatory effect in BV-2 cells and higher bioavailability in vivo	[52]
Curcumin-conjugated superparamagnetic iron oxide (SPIO) particles; magnetic nanoparticles	Curcumin (Cur-MNPs)	Co-localisation of particles with amyloid plaques, enabling less invasive (MRI-based) AD diagnosis	[53]
Gold and silver nanoparticles	Red ginseng water extract	Cytoprotective effect on neuron-like PC-12 cells due to decreased intracellular oxidative stress; promotion of synaptic impulse transmission	[59]
	Fresh ginseng leaf extract	Antioxidant, anticancer and anti-inflammatory activities in HaCaT cells, 3T3-L1 pre-adipocyte cells, A549 lung cancer and B16BL6 skin melanoma cancer cell lines and RAW 264.7 cell lines	[64]

**Table 2:** Nano-phytopharmaceuticals for therapeutic applications in CVDs.

Nanoformulation	Phyto-Pharmaceutical	Effects	References
Liposomes	Curcumin	Anti-hypercholesterolemic, anti-atherosclerotic and protective against cardiac ischemia and reperfusion.	[102]
PLGA nanoparticle	Quercetin	Anti-hypercholesterolemia, better cell rescue by lowering oxidized thiols and sustaining superior ATP production, improved therapeutics for ROS-based cardiac diseases.	[89]
Solid lipid Nanoparticle	Resveratrol	Protective action of vascular walls towards oxidation, inflammation, platelet oxidation and thrombus formation	[103,104]
1,2-diacyl-Sn-glycero-3-phosphocholine [EPC] and 1,2-dipalmitoyl-Sn-glycero-3-phosphocholine (DPPC) liposomes	Magnolol	Enhanced inhibitory effect on migration and hyperplasia of vascular smooth-muscle cells; Anti-platelet, anti-thrombotic, and anti-hypertensive via inhibiting MAPK family activation, Akt/ERK1/2/GSK3 β-catenin pathway, and angiotensin-converting enzyme (ACE)/angiotensin II (Ang II)/Ang II type 1 receptor (AT-1R) cascade and upregulating PPAR-β/γ and NO/guanosine 3′,5′-cyclic phosphate/PKG.	[85,105,106]
Nano-micelles	Tilianin	Protective effects of cardiomyocytes by inhibiting inflammation and oxidative stress during myocardial ischemia-reperfusion injury	[99]
PEGylated nanostructured lipid carriers	Baicalin	Improved myocardial ischemia; beneficial roles against the initiation and progression of CVDs such as atherosclerosis, hypertension, myocardial infarction, reperfusion and heart failure	[100,101]
Liposomes	Berberine	Effect of protecting heart failure, hypertension, hyperlipidemia, insulin resistance, arrhythmias, and platelet aggregation.	[96,107]

**Table 3:** Nano-phytopharmaceuticals for therapeutic applications in GI disorders.

Nanoformulation	Phyto-Pharmaceutical	Enteropathogen/GI Cell Lines; IC_50_/MIC	Reference
Polymeric (EPO)	*Berberis vulgaris*	*Entamoeba histolytica*; 26 ppm	[187]
Polymeric (EPO)	*Curcuma longa*	*Entamoeba histolytica*; 19 ppm	[187]
Polymeric (Cerium oxide)	*Nelumbo nucifera*	Human colon cancer (HCT 116); 4.16 µg/mL	[187]
Cellulose (Ethyl)	*Garcinia mangostana*	*Helicobacter pylori*; 62.5 µg/mL	[187]
Metallic (Silver)	*Toxicodendron vernicifluum*	*Helicobacter pylori*; 18.14 µg/mL	[187]
Metallic (Silver)	*Toxicodendron vernicifluum*	*E.coli*; 8.12 µg/mL	[187]
Metallic (Gold)	*Tribulus terrestris*	*Helicobacter pylori*; 16.75 µg/mL	[187]

## Data Availability

Not applicable.
